# Twitter Discourse on Nicotine as Potential Prophylactic or Therapeutic for COVID-19

**DOI:** 10.1101/2021.01.05.21249284

**Published:** 2021-01-06

**Authors:** Ramakanth Kavuluru, Jiho Noh, Shyanika W. Rose

**Affiliations:** Division of Biomedical Informatics, Internal Medicine, 230E MDS Bldg, 725 Rose St, Lexington KY 40506.; Computer Science Department, Lexington, KY; Center for Health Equity Transformation and Department of Behavioral Science, College of Medicine, Lexington, KY.

## Abstract

**Objective:**

The low observed prevalence of smokers among hospitalized COVID-19 patients in certain cohorts has led to a hypothesis regarding nicotine’s therapeutic role in COVID-19 prevention and treatment. As new scientific evidence surfaces, premature conclusions about nicotine are rife in social media, especially unwarranted leaps of such associations to vaping and smoking. This study reports on the prevalence of such leaps and the nature of authors who are making them.

**Methods:**

We used a Twitter API subscription service to download tweets (n = 17,533) that match terms indicating nicotine or vaping themes, in addition to those that point to a prophylactic or therapeutic effect and COVID-19 (January-July 2020). Using a windowing approach, we focused on tweets that are more likely to convey the therapeutic intent. We hand-annotated these filtered tweets and built a classifier that identifies tweets that extrapolate a nicotine link to vaping/smoking. We analyzed the frequently used terms in author bios, top Web links, and hashtags of such tweets.

**Results:**

21% of our filtered tweets indicate a vaping/smoking-based prevention/treatment narrative. Our classifier was able to spot tweets that make unproven claims about vaping/smoking and COVID-19 with a positive predictive value of 85%. Qualitative analyses show a variety of ways therapeutic claims are being made and user bios reveal pre-existing notions of positive stances toward vaping.

**Conclusion:**

The social media landscape is a double-edged sword in tobacco communication. Although it increases information reach, consumers can also be subject to confirmation bias when exposed to inadvertent or deliberate framing of scientific discourse that may border on misinformation. This calls for circumspection and additional planning in countering such narratives as the COVID-19 pandemic continues to ravage our world.

## INTRODUCTION

The link between nicotine and COVID-19 has garnered significant attention in both scientific and news media communities. The low prevalence of current smokers among COVID-19 patients and immunomodulatory effects of nicotine are cited as evidence for therapeutic potential of nicotine for affected patients^1^. Although current smokers were found to be at a reduced risk of SARS-CoV-2 infection, meta-analyses reveal that former smokers were at an increased risk of hospitalization, severity, and mortality due to COVID-19^2^ while no such associations were found with current smoking status. However, there are several significant limitations of using electronic health record derived determination of smoking status (including missing data on smoking status and lack of adjustment for the demographic profile of those tested/admitted to the hospital), and no studies included in the meta-analysis verified smoking status with biochemical measures. There is at least one clinical trial planned by French researchers to assess nicotine’s therapeutic and prophylactic potential^3^. Overall, in current literature on these themes, authors clearly indicate that smoking’s severe addiction and conclusively harmful health effects dictate that it shouldn’t be used as a means to prevent/treat COVID-19. Scientific explorations are ongoing about nicotine and its role as a potential COVID-19 therapeutic in controlled settings^4^. In the meantime, it is important to understand how this so called “nicotine hypothesis” plays out in consumer discourse on social media regarding beliefs and attitudes towards tobacco products (in the context of the ongoing pandemic).

In this paper, we examine publicly available tweets that discuss nicotine or vaping as preventative or treatment options for COVID-19 with associated extrapolations to smoking/vaping’s therapeutic potential in retrieved messages. To our knowledge, there are only two recent efforts that seemed to have touched upon the themes of our study: the first offers an anecdotal indication of unsubstantiated health claims^5^ and does not focus on the nicotine hypothesis and the latest effort examines sentiment surrounding vaping and smoking in the context of the pandemic^6^; this latter effort’s focus on sentiment has a different purpose of general perceptions of people and not about quantifying misinformation involving vaping/smoking. Our effort has a deliberate focus on unproven extrapolations of the nicotine hypothesis for COVID-19 spanning tweets from a seven-month duration.

## METHODS

We downloaded ALL publicly available tweets authored between Jan 1, 2020 and July 31, 2020 containing **at least one term** (or associated hashtag) from each of the three following groups:

1. nicotine, e-cigarette(s), ecig(s), vaping;*
2. prevent(s), prevented, preventing, prevention, preventative, preventive, prophylactic, prophylaxis, precaution(ary), treat(s), treated, treating, treatment, cure(s), cured, curing;
3. covid, coronavirus, covid19, corona, covid_19, covid-19, SARS-Cov-2.

We focused on having at least one term from each group because we wanted to explore discussions that are portraying nicotine and associated products as helpful in dealing with COVID-19. Before we proceed, we note that the IRB at the University of Kentucky deemed that this type of research does not meet the definition of human subjects and thus does not require additional IRB review based on these two criteria: 1. The data is publicly available; and 2. There is no interaction or intervention with subjects. Nevertheless, we only report aggregate metrics (e.g., counts, proportions) and show very few example tweets without author attributions that convey some viewpoints.

Our conjunctive Twitter query outlined earlier in this section led to a total of 17,533 tweets of which 16,064 were in English. We observed several of these tweets were discussing vaping bleach (misinformation propagated by a radio talk show host) for treating COVID-19 and hence we removed all tweets containing “bleach”. This further pruned the dataset to 13,718 tweets. Among these, we imposed a strict constraint that all three terms (one each from three groups above) must occur within a window of six consecutive words (three additional words on top of the three required terms) after commonly used stops words^†^ are removed from the tweet. This was needed to weed out tweets that contain the three terms but are separated by many other terms and whose overall meaning is something quite different from our search intent. For example, consider the tweet by a public health organization in Oregon (USA): “Smoking and **vaping** involve hand-to-mouth contact that may make it easier to spread **COVID-19** to the user and other surfaces**. Prevent** the spread of **COVID-19**, quit today.” Although a term from each group in our query appears here, they are farther apart and do not convey the intent of our search. The six consecutive word window constraint removes this tweet from our data as it does not satisfy it. This window constraint resulted in a smaller subset of 5192 tweets, which is henceforth called the filtered dataset. From this subset, we (first and second author together) annotated a set of 400 tweets randomly selected to identify if the tweets are actually discussing potential prevention or treatment aspects of nicotine for COVID-19. Among such tweets, we additionally identified those tweets that were extrapolating the nicotine connection to vaping or smoking with direct or implicit claims about vaping or smoking being beneficial in the context of COVID-19. A supervised machine learned model^7^ was built and evaluated using cross-validation to identify these tweets that resort to this type of extrapolation based on tweet text and associated user name. The specific model^7^ is based on association rules that link presence of certain key words to classes (extrapolating tweet or the negative class). Subsequently, this model was applied to the remaining unannotated filtered dataset of tweets to identify tweets that extrapolated nicotine-related news to smoking and vaping. We ranked top 20 frequent words in the personal bios of Twitter users who tweet such extrapolating messages as opposed to users in the rest of the tweets. This is to see if users who tweet such messages had pre-existing viewpoints that may have led them to extrapolate nicotine research to smoking or vaping. We also looked at top (most frequent) Web links and hashtags used in extrapolating tweets and the remaining tweets. This was done by just counting the tweets containing each of the unique URLs and hashtags in the dataset.

## RESULTS

Out of the 400 annotated from the 5192 filtered tweets, 337 turned out to be conveying a treatment/preventative aspect leading to a positive predictive value (PPV) of 84.25% with 95% confidence interval [80.22%, 87.60%]. Of the 337 tweets, we also found 84 to be linking nicotine with smoking/vaping as an extrapolation of research surrounding nicotine or (more rarely) discussing smoking/vaping directly as being therapeutic. This translates to 84/400 = 21% (95% confidence interval: [17.18%, 24.39%]) tweets making such claims. Some pertinent examples from our dataset are shown in Table 1 demonstrating the variety of takes on the controversial topic. We only displayed examples here where the tweet contents cannot be linked back to a real person by searching on Twitter. That is, the user names (and display names) corresponding to messages in Table 1 and other illustrations in this paper can only be traced back to personalities not using their real name (e.g., @JohnnyVapes). However, we want to paraphrase examples of this nature that come from accounts with proper names (and websites of the authors) without displaying the tweet verbatim. In one instance, a tweeter with over 10,000 followers wondered if vaping was the only thing that can cure COVID-19. In another case, a physician tweeter (with over 2000 followers) from a prestigious U.S. Carnegie R1 university states the facts appearing in a linked news article about the French nicotine clinical trial but also includes a “smoking protects” phrasing in the message that is unwarranted and prone to misinterpretation. Another doctoral level researcher (with over 5000 followers) holding a scientific role in a non-profit for smoking cessation takes a nuanced approach that does not outright claim vaping-driven prevention conclusively but still indicates that there exists evidence supporting it.

The classifier that was built to identify the tweets that involve vaping or smoking (shown in Table 1) as potential aids to handle COVID-19 had a PPV of 85% with 65% sensitivity. When it was applied to the filtered dataset, 576 were identified as such, translating to over 11% (576/5192) of the dataset. This proportion is smaller than 21% observed in the human annotated sample because the classifier’s sensitivity is only 65%. We preferred the classifier to be more focused on high precision (PPV) because we wanted to look at the bio text of the Twitter profiles that tweet favorably, involving smoking or vaping. Outside that purpose of separating bios of extrapolating users, the classifier was not thus applied for deriving general proportions.

Bios are brief descriptions that Twitter users post on their profiles typically talking about themselves. Table 2 shows a distribution of top bio words that occur in profiles of users of the 576 tweets that were classified as expressing a favorable view of vaping or smoking for COVID-19 in juxtaposition with those from authors of remaining tweets. The words in the table are sorted in descending order in terms of numbers of bios in that group containing them. The percentages in the parentheses in the table correspond to the proportion of unique users whose bio contains the term. A sample bio of one of the favorable view authors reads: “#Vaping ends #smoking harms. Advocate #HarmReduction. Stop the #VapeBan. Deflate nannycrat pseudoscience masquerading as “#publichealth” #AbolishFDA”. It is quite evident from Table 2 that “advocate” and “vaping” feature at the very top in the bios of such authors. Words “advocate” and “vaping” show up in 1.7% and 1.6% of authors in the *other* group, while featuring in 6.3% and 5.7% of authors in favorable view group, respectively, clearly indicating over 3*X* increase in the latter group.

We also considered a different way of analyzing users generating the vaping/smoking tweets. We begin with a simple definition that any user bio or username containing the following terms as substrings is “pro-vaping”: *smokefree, “smoke free”, “harm reduction”, harmreduction, e-cigarette, ecigarette, vape, vapor, vapour, vaping, ecig, eliquid, ejuice, e-liquid, tobaccofree, tobacco-free, “tobacco free”, thr* (acronym for tobacco harm reduction). Note that because we use substrings, bios containing terms such as *vape*r, #We*Vape*WeVote, #*Vaping*SavesLives, #*Vaping*Advocate will all match our condition. With this definition, 6.8% of tweeters in the filtered dataset are pro-vaping but they generated a total of 12.6% of its tweets. They also author 21.2% of the 576 smoking/vaping tweets.

## DISCUSSION

21% of tweets matching our windowing constraint also express a leap from a nicotine-COVID-19 link to potential benefits of smoking/vaping, even if it is implied. This is clearly an alarmingly non-trivial proportion. Examples from Table 1 and other paraphrased instances show the variety of such tweets ranging from indirect/subtle hints to more explicit declarations. When our classifier was run on unlabeled filtered dataset to identify all such tweets (along with those in the annotated dataset), the corresponding bios reveal vaping related terms featuring at the top (from Table 2). Additionally, results based on the pro-vaping user definition showed that despite constituting less than 7% of users, they authored over 20% of smoking/vaping tweets. This leads us to believe that the tweets that are extrapolating a potential nicotine-COVID-19 link to smoking/vaping as being therapeutic are authored by those having pre-existing positive views on vaping or smoking. But, there is potential for casual Twitter users to be exposed to these types of messages providing potentially biased interpretations of scientific findings. This might encourage them to continue their tobacco product consumption habits or initiate new habits, both possibilities that may lead to negative consequences for public health overall. The first column of Table 2 also shows an identification with the current president of the U.S. (Trump) and his campaign slogan (MAGA) potentially indicating associations with political affiliations.

The most frequently used Web link in the favorable group is a news media piece titled: “This new evidence shows nicotine might prevent smokers from contracting the coronavirus” making an indirect link to smoking. The top link in the other group is a news piece titled: “France testing whether nicotine could prevent coronavirus”. It is straightforward to see this link is positing more of a hypothesis while the former one claims evidence why smokers may be protected from the virus. Among the top ten links in both groups, there are only two links that directly refer to published peer-reviewed research articles^1,8^ both in the favorable view group.

Upon examining the top ten frequently used hashtags in both groups, we did not find much difference except for one interesting tag #QuitLying that only shows up in the favorable view tweets. This hashtag is often used in replying to tweets by other users who may be tweeting about the potential risks of COVID-19 for smokers and vapers. For instance, consider this tweet: “There’s compelling evidence that #nicotine inhalation prevents #COVID19 infection and reduces the severity of symptoms. Whatever you’re #smoking, it’s clearly addled your brain. #QuitLying”. This was used as a reply to another tweet by a verified medical doctor who tweeted: “Thanks to everyone who joined the @voxmedia, @TobaccoFreeKids + @Bloombergdotorg panel on tobacco use and #COVID19. With over 5M high school students using e-cigarettes, there’s never been a more urgent moment to talk about the connection between tobacco use and the virus”. This hashtag appears to be a mechanism to engage with others who might be having a non-favorable perspective of tobacco products or nicotine for COVID-19.

Before we conclude, we outline some limitations: only public English language tweets were analyzed; so the findings cannot be generalized to the entire conversation about COVID-19 and tobacco use on all social networks especially on platforms like Facebook and WhatsApp where most of the messaging is not public. The discussion is likely evolving as the pandemic changes and this only captures discussion during the specific duration of January to July in 2020.

Overall, our results demonstrate that there is a nontrivial amount of chatter on Twitter that promotes the not-yet-validated nicotine hypothesis for COVID-19 which at times is also extrapolated to vaping and smoking, spreading false hope to tobacco product consumers without conclusive evidence. Also given evidence of increased risk of adverse outcomes from COVID-19 among smokers compared with non-smokers, social media messages conflating the ‘nicotine hypothesis’ with potential benefits of smoking for COVID-19 may be detrimental to smokers^9^. On Twitter, studies have shown that false news stories reach significantly faster and farther than true ones^10^. Recent findings also show that exposure to misinformation that smoking helps with COVID-19 actually leads to an increase in tobacco consumption^11^. Hence, federal health agencies, healthcare organizations, physicians should all be extremely vigilant in communicating verified information to the general public and patients in the wake of nicotine related misinformation about COVID-19. Additionally, engaging credible voices and approaches to provide balanced information and combat misinformation about vaping and smoking and COVID 19 to the public is needed as trust of typical sources of information (e.g., FDA) may also be weakened through this discourse^12^.

## Figures and Tables

**Table 1:** Tweets that appear to indicate smoking or vaping as being protective for COVID-19

Example tweet	Comments
*“****Cigarettes*** *can keep coronavirus away? Researchers test if* ***nicotin****e could* ***prevent COVID-19****.”* ***Smokers****, rejoice! Celebrate by light up*.	This message clearly extrapolates a potential nicotine-COVID link to cigarettes, encouraging smokers to continue their habit. The quoted text is the title of a news article that discusses the potential nicotine link to COVID-19.
*liberals don’t want you to know that* ***coronavirus*** *can be* ***cured*** *by* ***vaping*** *mango juul pods! mr. trump reverse the ban people are dying!*	This is a message that states that vaping Juul pods can cure the infection and appeals to the U.S. president at the time to reverse the ban (on flavored vaping products)
*No doubt that* ***vaping*** *could have* ***prevented*** *a multitude of* ***Covid19*** *deaths. PG, the main ingredient in Eliquid was found to kill the flu virus (among other microbial viruses) which means it could have prevented individuals from contracting the virus*	This message directly talks about vaping as a prevention method without reference to nicotine. It also includes a screenshot of a blog post on vapemate.co.uk (a commercial tobacco product vendor) that discusses some benefits of propylene glycol as a germicide.
*WHOA!!* ***NICOTINE*** *PREVENTS #COVID19 INFECTIONS!! I always knew* ***smoking*** *would eventually save lives* *This new evidence shows* ***nicotine*** *might* ***prevent*** *smokers from catching coronavirus:* *https://on.mktw.net/3541YGO*	A direct extrapolation of a potential nicotine connection to smoking without any justification or evidence.
*What if you had a choice between a 5.6% risk of dying in the next few weeks vs. a 33% chance of acquiring a nasty disease in your 80s? That’s the risk balance when it comes to #covid19 and #smoking. The evidence is clear and overwhelming that* ***#nicotine prevents #coronavirus*** Self-reply*: But the good news is you don’t have to choose! There’s a #harmless and pleasurable way to enjoy* ***#nicotine*** *to reduce your risk of getting* ***#COVID19*** *by at least a factor of 2 and possibly as much as 80x. Get yourself a #vape now!*	This tweeter, with over 5000 followers, self identifies as a tobacco influencer (in their bio) and quote-tweets another thread of tweets indicating low prevalence of smoking among COVID-19 patients. The tweet compares the case fatality rate with some other potential future ailment due to smoking at a much older age, setting up a false equivalency. It declares a nonexistent causal link between nicotine and SARS-Cov-2. Next message in the thread encourages vaping citing a preprint that is not peer-reviewed and is based only on self-reported diagnoses of COVID-19.

**Table 2:** Top 20 bio words sorted by proportion of unique users authoring the tweets belonging to each group (extrapolating vs rest); **bold** words are tobacco product related.

Top bio words for authors of tweets that appear to promote a favorable view of smoking or vaping for COVID-19	Top bio words for authors of remaining tweets satisfying the window constraint
Unique users: 508	Unique users: 4045
**Advocate** (6.3%)	Love (4.4%)
**Vaping** (5.7%)	News (3.3%)
**Harm** (3.9%)	Life (3.3%)
Love (3.7%)	Health (2.5%)
News (3.5%)	World (2.2%)
**Reduction** (3.3%)	Like (2.2%)
Trump (3.1%)	Views (2.2%)
**Tobacco** (3.1%)	Fan (2.1%)
**Free** (3.1%)	Tweets (2.1%)
People (2.8%)	Music (2.0%)
MAGA (2.6%)	**Free** (1.9%)
Lover (2.6%)	Politics (1.8%)
Writer (2.6%)	Follow (1.8%)
Fan (2.4%)	People (1.8%)
Since (2.4%)	**Advocate** (1.7%)
Media (2.4%)	Lover (1.7%)
**Smoking** (2.2%)	Father (1.6%)
**Vaper** (2.2%)	Writer (1.6%)
**Smoke** (2.2%)	**Tobacco** (1.6%)
Truth (2.2%)	**Vaping** (1.6%)

## Highlights:

- Social media plays an increasingly critical role in tobacco product related communication of both objectively assessed information and biased disinformation.
- Low prevalence of smokers among hospitalized COVID-19 patients is leading to a rapidly evolving scientific discourse on the role of nicotine for COVID-19. It is unclear how this scientific landscape is being interpreted and communicated on social media.
- This paper demonstrates that a nontrivial portion of chatter surrounding nicotine’s role in COVID-19 extrapolates an unproven nicotine hypothesis to vaping and smoking.
- This favorable view of vaping/smoking appears to be mostly carried out by players with pre-existing positive stances regarding the role of vaping in tobacco prevention.
- Our findings warrant vigilance on part of public health agencies and healthcare professionals as they communicate accurate and objective findings at the intersection of nicotine and COVID-19 to counter disinformation.

## Footnotes

* Most of the sample tweets we downloaded that exclusively mentioned smoking or cigarettes without references to nicotine are genuinely highlighting the dangers of smoking during COVID-19. In light of this and cost effectiveness of our Twitter API subscription to obtain an exhaustive dataset of tweets, we left out “smoking”, “cigarettes”, and “tobacco”. Our goal was to study the chatter surrounding the nicotine mentions.

† http://ir.dcs.gla.ac.uk/resources/linguistic_utils/stop_words

## References

[1] Farsalinos K, Barbouni A, Niaura R. Systematic review of the prevalence of current smoking among hospitalized COVID-19 patients in China: could nicotine be a therapeutic option? Internal and Emergency Medicine 2020:1–8. 32385628 10.1007/s11739-020-02355-7 PMC7210099

[2] Simons D, Shahab L, Brown J, The association of smoking status with SARS-CoV-2 infection, hospitalisation and mortality from COVID-19: A living rapid evidence review. Qeios 2020 10.1111/add.15276 PMC7590402 33007104

[3] Changeux J-P, Amoura Z, Rey FA, A nicotinic hypothesis for Covid-19 with preventive and therapeutic implications. Comptes Rendus Biologies 2020;343(1):33–39. 32720486 10.5802/crbiol.8

[4] Tizabi Y, Getachew B, Copeland RL, Nicotine and the nicotinic cholinergic system in COVID 19. The FEBS Journal 2020 10.1111/febs.15521 PMC7436654 32790936

[5] Majmundar A, Allem J-P, Cruz TB, Public health concerns and unsubstantiated claims at the intersection of vaping and COVID-19. Nicotine & Tobacco Research 2020 10.1093/ntr/ntaa064 PMC7184377 32285129

[6] Kamiński M, Muth A, Bogdański P. Smoking, Vaping, and Tobacco Industry During COVID-19 Pandemic: Twitter Data Analysis. Cyberpsychology, Behavior, and Social Networking 2020 10.1089/cyber.2020.0384 32757951

[7] Integrating classification and association rule mining. Proceedings of the Fourth International Conference on Knowledge Discovery and Data Mining; 1998.

[8] Tindle HA, Newhouse PA, Freiberg MS. Beyond smoking cessation: Investigating medicinal nicotine to prevent and treat COVID-19. Nicotine & Tobacco Research 2020 10.1093/ntr/ntaa077 PMC7239141 32383751

[9] Farsalinos K, Barbouni A, Poulas K, Current smoking, former smoking, and adverse outcome among hospitalized COVID-19 patients: a systematic review and meta-analysis. Therapeutic advances in chronic disease 2020;11:2040622320935765. 32637059 10.1177/2040622320935765 PMC7318805

[10] Vosoughi S, Roy D, Aral S. The spread of true and false news online. Science 2018;359(6380):1146–51. 29590045 10.1126/science.aap9559

[11] Luk TT, Zhao S, Weng X, Exposure to health misinformation about COVID-19 and increased tobacco and alcohol use: a population-based survey in Hong Kong. Tobacco control 2020 10.1136/tobaccocontrol-2020-055960 32855353

[12] Freckelton QI. COVID-19: Fear, quackery, false representations and the law. International Journal of Law and Psychiatry 2020:101611. 10.1016/j.ijlp.2020.101611 PMC7351412 32911444

